# Primary Eccrine Porocarcinoma of the Breast: A Case Report and Review of Literature

**DOI:** 10.1155/2022/4042298

**Published:** 2022-05-31

**Authors:** Yi Xin Li, Mihir Gudi, Zhiyan Yan

**Affiliations:** ^1^Duke-NUS Medical School, Singapore; ^2^Department of Pathology and Laboratory Medicine, KK Women's and Children's Hospital, Singapore; ^3^Breast Department, KK Women's and Children's Hospital, Singapore

## Abstract

Eccrine porocarcinoma (EPC) is a rare cutaneous neoplasm, with less than 500 reported cases worldwide since it was first described in 1963. EPC tends to affect the elderly and most commonly affects the head and neck. The mainstay of EPC treatment is surgery, with lymphadenectomy in the case of nodal involvement or presence of unfavourable characteristics. No evidence exists to guide the use of adjuvant chemotherapy or radiation. EPC is prone to misdiagnosis given its multiple clinical and histopathological mimics, especially in uncommon sites of presentation such as the breast. Herein, we report the case of a 59-year-old woman who presented with a left breast skin lump. The biopsied specimen revealed an infiltrative carcinoma involving the dermis and epidermis with positive IHC staining for P63 and CK5/6 and negative staining for ER, PR, and HER2. The tumour was resected and diagnosed as EPC with atypical features as overlapping characteristics of squamous cell carcinoma (SCC) were detected on histopathological analysis. In our case, a simple mastectomy with broad margins and axillary lymph node dissection with adjuvant radiotherapy to a dose of 60 Gy failed to achieve loco-regional control with nodal recurrence occurring 4 months postsurgery—a testament to the aggressive course of this rare malignancy.

## 1. Introduction

Eccrine porocarcinoma is a rare, invasive, and highly aggressive cutaneous tumour originating from the intraepidermal eccrine sweat duct (acrosyringium), first described by Pinkus and Mehregan in 1963 [1]. Its exact prevalence is unknown but estimated to constitute 0.005% to 0.01% of all skin cancers [2]. EPC has no predisposition for ethnicity or gender and tends to occur in older people in the sixth to seventh decade of life although there is a wide distribution of presenting ages ranging from 7 months to 97 years [3]. Surgical resection remains the mainstay treatment of choice by general consensus with limited evidence on the benefit of adjuvant radio- or chemotherapy [4]. To our knowledge, only 3 cases of primary EPC in the breast have been reported [4–6]. We present a rare case of primary breast EPC in a 59-year-old woman with lymph node metastasis and discuss the clinical presentation and histopathological findings and review the pertinent literature.

## 2. Case Report

**Figure 1:**
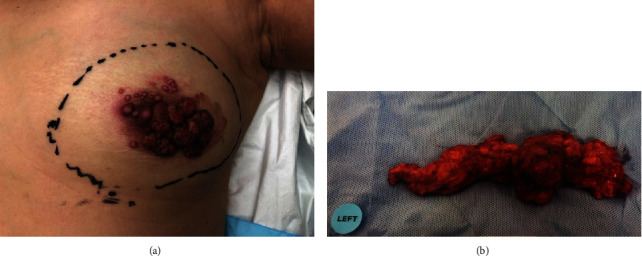
(a) Clinical appearance of the breast lesion. (b) Clinical appearance of the axillary dissection specimen.

A 59-year-old woman with a positive family history of breast cancer presented with a superficial left breast lump of 5 months duration without nipple discharge. Her preexisting medical conditions were hypertension, dyslipidemia, and diabetes. Examination revealed a 3 × 3 cm raised polypoid lesion at the left periareolar region with palpable left axillary lymphadenopathy (Figure 1(a)).  Figure 1(b) shows the axillary lymph node dissection specimen.

**Figure 2:**
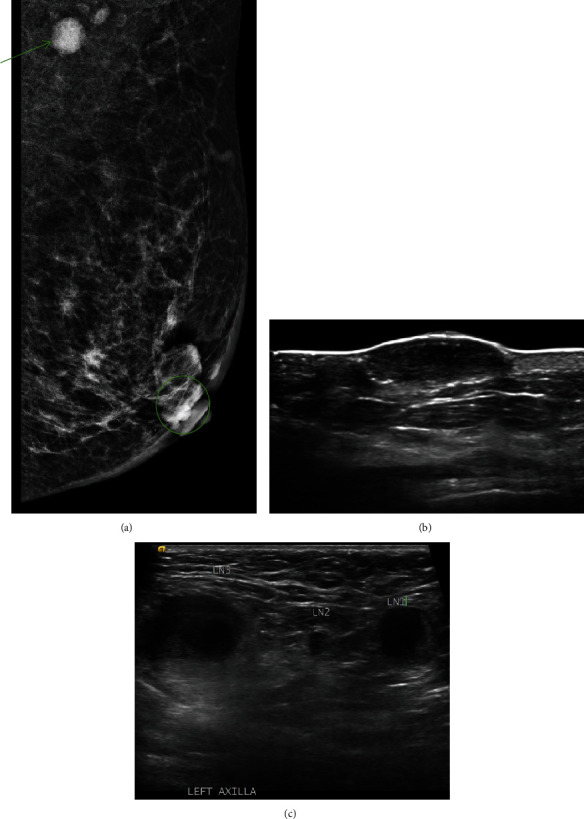
(a) Mediolateral oblique mammogram of the left breast showing a circumscribed periareolar opacity (circle) and dense axillary lymph node (arrow). (b) Superficial hypoechoic nodule on left breast sonography. (c) Lymphadenopathy on left axilla sonography.

Bilateral mammography revealed a 3.7 cm circumscribed opacity at the left periareolar region (Figure 2(a)), while ultrasonography showed a 3.3 × 3.0 × 0.9 cm oval hypoechoic mass (Figure 2(b)) with left axilla lymphadenopathy (Figure 2(c)). Punch biopsy demonstrated an infiltrative carcinoma involving the dermis and epidermis. Immunohistochemical (IHC) staining was P63 and CK5/6 positive and mammaglobin, estrogen receptor (ER), progesterone receptor (PR), and HER-2 negative. Core biopsy confirmed positive nodal involvement while Computed Tomography (CT) imaging did not detect metastasis. These findings suggested a primary skin cancer instead of breast carcinoma.

Our patient underwent a simple mastectomy with 4 cm gross margins as well as an axillary lymph node dissection up to level II (Figure 1(b)). Macroscopically, the tumour was continuous with the epidermis with subcutis and breast parenchyma invasion; its maximal diameter and thickness were 6 cm and 1.3 cm, respectively. 12 out of 17 lymph nodes were positive for metastasis with extranodal extension present in several involved nodes. The largest nodal metastatic focus measured about 4.5 cm. The surgical margins were tumour-free.

**Figure 3:**
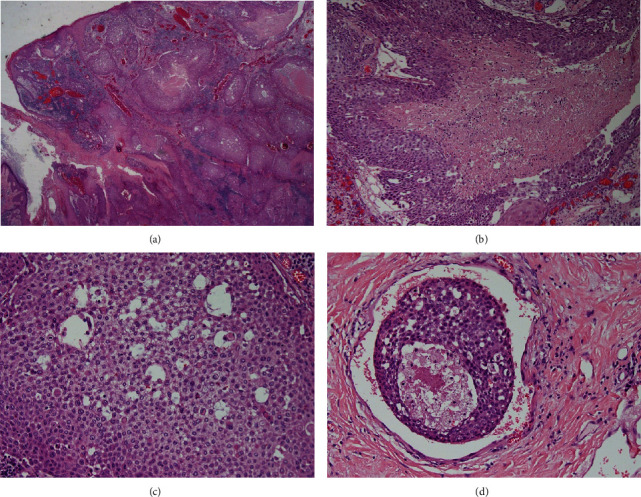
Tumour H&E sections: (a) skin-based carcinoma showing islands of infiltrating tumour (×40); (b) focus of central necrosis (×100); (c) cytoplasmic clearing within tumour islands with nuclear pleomorphism (×100); (d) focus of lymphovascular invasion (×200).

**Figure 4:**
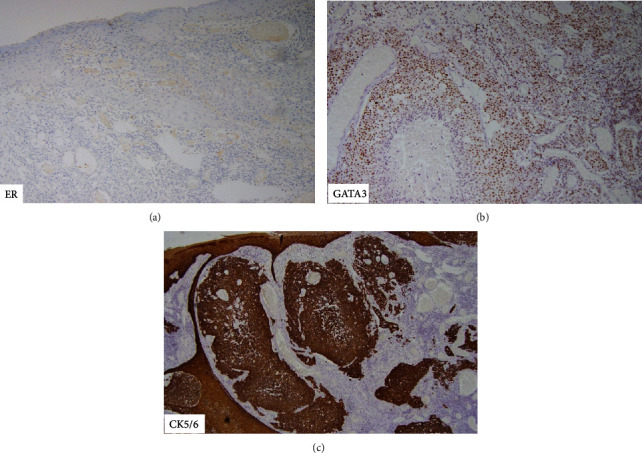
Immunohistochemistry analysis results: (a) ER (-); (b) GATA 3 (+); (c) CK5/6 (+).

Histologically, the tumour was poorly differentiated, consisting of infiltrative islands (Figure 3(a)) of solid sheets, nests, and cords of rounded to polygonal cells surrounded by desmoplastic stroma. Areas of necrosis (Figure 3(b)) and keratinisation were observed together with nuclear pleomorphism and conspicuous mitoses (Figure 3(c)). Lymphovascular invasion was identified (Figure 3(d)). Ductal and glandular structures were not identified. IHC staining was negative for CK7, CEA, ER (Figure 4(a)), PR, and HER2, focally positive for EMA, P16, and GATA3 (Figure 4(b)), and diffusely positive for CK5/6 (Figure 4(c)) and p63.

The diagnosis of EPC with atypical features of SCC was made, and subsequently, the patient underwent 6 weeks of adjuvant radiotherapy (60 Gy in 30 fractions) to the left axilla and chest wall 2 months postsurgery. The dose and fractionation were extrapolated from treatment guidelines for head and neck SCC given that SCC features were present in this case [7]. However, CT imaging detected nodal recurrence 2 days before the last dose of radiotherapy. The patient rejected reexcision and palliative chemotherapy and is managed supportively at 1-year follow-up.

## 3. Discussion

EPC is prone to misdiagnosis given its variable presentations. Clinically, EPC can present as ulcerating polypoid, exophytic growths, or verrucous plaques and be misdiagnosed as SCC, Bowen's disease, seborrheic keratosis, or pyogenic granuloma; histopathologically, EPC also has to be distinguished from basal cell carcinoma, hidradenocarcinoma, and most importantly SCC [5].

In our case, additional clinical mimics such as an invasive mammary ductal carcinoma had to be considered as well. However, triple assessment pointed towards a primary skin neoplasm instead of breast carcinoma. Differentials were narrowed to either a sweat gland tumour or squamous cell carcinoma (SCC), its main histologic mimic. On histological analysis, the absence of visible ductal lumina is uncharacteristic of EPC while the remarkably uniform “poroid” appearance of tumour cells is highly unusual for SCC. IHC staining result was equivocal for either diagnosis although negative CK7 and CEA staining is uncommon in EPC [8]. This atypical case demonstrates the utility of identifying additional IHC stains to differentiate EPC from SCC (e.g., S-100, CD117, CK19, and nestin) given their treatment differences which impact prognosis and survival [9, 10]. On the overall, given the tumour's morphology which was more suggestive of EPC instead of SCC, our patient was diagnosed with EPC and the diagnosis is supported by the tumour's highly aggressive nature given its early recurrence despite intensive treatment.

The etiology behind EPC development is unknown. Suggested risk factors include exposure of skin to burns, trauma or radiotherapy, immunosuppression, and prolonged exposure to ultraviolet light which were all absent in our patient [11]. EPC can arise de novo or via malignant transformation of a benign eccrine poroma usually over many years [12]. Reportedly, up to 18% to 50% of EPCs degenerate from initially benign poromas [8]. Unlike poromas which primarily present in regions with high densities of eccrine sweat glands (palmoplantar regions), EPC occurs rarely in these regions and more frequently at the head and neck followed by lower extremities [3]. However, EPC can affect any body part, and to our knowledge, only 3 cases of primary breast EPC have been reported [4–6]. An overview of these patient characteristics and treatment outcomes are summarised in Table 1 which largely correlates with our understanding of EPC presentation and aggressive natural history. The histopathological and immunohistochemical features in these cases were also typical unlike in our case where diagnosis of EPC was not as straightforward.

There is no standard guideline for EPC treatment. However, the first-line approach by consensus is surgical resection either with Mohs Micrographic Surgery (MMS) or more commonly a Wide Local Resection (WLE) with broad margins (>2 cm), without which prognosis and survival rates are significantly poorer [13]. The curative rate of WLE is reportedly 80% with a 20% probability of local or nodal recurrence despite negative excision margins as realised in our case [13].

The benefit of adjuvant radio and chemotherapy in EPC remains unproven. Le et al. reported that adjuvant radio or chemotherapy did not significantly improve prognosis or overall survival in their meta-analyses of 120 EPC head and neck cases [14]. However, there may be a role for adjuvant radiotherapy in EPC with positive resection margins or presence of poor prognostic features (as “infiltrative” or “pushing” subtypes of EPC, >14 mitoses per high-power field, lymphovascular invasion, and tumoural involvement > 7 mm) as in our case, given the higher risk of recurrence and metastasis [15, 16]. In cases of recurrent and/or metastatic EPC, individual reports have demonstrated the possibility of partial or complete remission with adjuvant chemotherapy such as docetaxel and 5-fluorouracil [6, 17, 18]. Unconventional treatments have also been utilised in recurrent and/or metastatic EPC such as immunotherapy (interferon alpha, interleukin 2, and pembrolizumab), isotretinoin, electrochemotherapy, intralesional photodynamic therapy, CyberKnife radiosurgery, and hormonal treatment (tamoxifen) in the case of positive ER/PR receptors with variable success [19–25].

Recent research may shed light on the high rates of recurrence in EPC even with adjuvant treatment—Taghizadeh-Hesary et al. highlighted the importance of mitochondria's role in cancer metabolism such as mediating efflux pump expression and scavenging reactive oxygen species (ROS) which confers resistance to chemo- and radiotherapy [26]. A stronger functional mitochondrial status may explain why some cancers such as EPC are highly recurrent and resilient. Further research into antimitochondrial therapy may thus have potential in improving treatment outcomes for these cancers.

## 4. Conclusion

EPC is a rare and aggressive skin neoplasm with a high risk of misdiagnosis due to multiple clinical and histopathological mimics. It has a high propensity for dermal lymphatic invasion which leads to frequent nodal involvement and distant metastasis with a high risk of recurrence. A multidisciplinary approach towards diagnosis and early intervention with frequent follow-up surveillance is therefore essential in pursuit of complete remission.

## Figures and Tables

**Table 1:** General characteristics, EPC histopathological and immunohistochemical features, treatment modalities, and outcomes in terms of response or survival.

Age/sex	Site and largest dimension (mm)	Lymph node/distant metastasis	Histopathological and IHC features of EPC	Treatment received	Treatment outcome	First author and year
92/F	Right breast, 60 mm	+	n.a.	Surgery (mastectomy)	Palliative care, adjuvant therapy rejected	Bonito (2020)
74/F	Right breast, 45 mm (first recurrence)	—	Infiltrative subtype with squamous differentiation and lymphovascular invasion Negative postop margins IHC positive for p63, CK5, and EMA and negative for ER and PR	Surgery (WLE) followed by reexcision (WLE) and adjuvant RT (66 Gy)	Overall survival of 3.3 years; first recurrence 22.5 months after initial resection and patient died 17.5 months after reexcision and adjuvant RT	Morten (2018)
54/M	n.a.	+	Unknown subtype IHC positive for AE1/AE3 and CEA and negative for S-100 and CK5/6	Surgery (WLE) followed by chemotherapy (cisplatin, 5-fluorouracil, and docetaxel) and reexcision (WLE)	Local recurrence and metastasis 1 year postop; complete remission achieved after docetaxel chemotherapy	Aaribi (2013)

M: male; F: female; n.a.: not available.

## Data Availability

Data sharing not applicable to this article as no datasets were generated or analysed for this study.
